# Vaccine adverse event reporting system (VAERS): Evaluation of 31 years of reports and pandemics’ impact

**DOI:** 10.1016/j.jsps.2022.10.001

**Published:** 2022-10-07

**Authors:** Ohoud A. Almadani, Thamir M. Alshammari

**Affiliations:** a Saudi Food and Drug Authority, Riyadh, Saudi Arabia; b Medication Safety Research Chair, King Saud University, Riyadh, Saudi Arabia; c Colleage of Pharmacy, Prince Sattam bin Abdulaziz University, Alkharj, Saudi Arabia

**Keywords:** VAERS, AEFI, AESI, Surveillance, Vaccines, COVID-19

## Abstract

**Background:**

Vaccine adverse event reporting system (VAERS) was established in the United States (U.S.) as an early warning system with a main purpose of collecting post-marketing Adverse events following immunizations (AEFIs) reports to monitor the vaccine safety and to mitigate the risks from vaccines. During the coronavirus diseases 2019 (COVID-19) pandemic, VAERS got more attention as its important role in monitoring the safety of the vaccines. The aim of this study was to investigate VAERS patterns, reported AEFI, vaccines, and impact of different pandemics since its inception.

**Methods:**

This was an observational study using VARES data from 2/7/1990 to 12/11/2021. Patterns of reports over years were first described, followed by a comparison of reports statistics per year. Furthermore, a comparison of incidents (death, ER visits, etc.) statistics over years, in addition to statistics of each vaccine were calculated. Moreover, each incident's statistics for each vaccine were calculated and top vaccines were reported. All analyses were conducted using R (Version 1.4.1717) and Excel for Microsoft 365.

**Results:**

There were 1,396,280 domestic and 346,210 non-domestic reports during 1990–2021, including 228 vaccines. For both domestic and non-domestic reports, year of 2021 had the highest reporting rate (48.52 % and 70.33 %), in addition a notable change in AEFIs patterns were recorded during 1991, 1998, 2000, 2006, 2009, 2011, and 2017. AEFIs were as follow: deaths (1.00 % and 4.08 %), ER or doctor visits (13.37 % and 2.27 %), hospitalizations (5.84 % and 27.78 %), lethal threat (1.42 % and 4.38 %), and disabilities (1.4 % and 7.96 %). Pyrexia was the top reported symptom during the past 31 years, except for 2021 where headache was the top one. COVID-19 vaccines namely Moderna, Pfizer-Biontech, and Janssen were the top 3 reported vaccines with headache, pyrexia, and fatigue as the top associated AEFIs. Followed by Zoster, Seasonal Influenza, Pneumococcal, and Human papillomavirus vaccines.

**Conclusions:**

The large data available in VARES make it a useful tool for detecting and monitoring vaccine AEFIs. However, its usability relies on understating the limitations of this surveillance system, the impact of governmental regulations, availability of vaccines, and public health recommendations on the reporting rate.

## 1 Introduction

Vaccine is a substance intended to boost the body's immunological defenses against diseases (Centers for Disease Control and Prevention, 2021). The Council for International Organizations of Medical Sciences (CIOMS) defined an adverse event following immunization (AEFI) as “any untoward medical occurrence which follows immunization, and which does not necessarily have a causal relationship with the usage of the vaccine. AEFI may be any unfavorable or unintended sign, abnormal laboratory finding, symptom or disease” (World Health Organization (WHO), 2014). To identify these AEFIs, vaccine testing for safety is conducted in laboratories on animals and humans in clinical trials, similar to medications’ approval process (Centers for Disease Control and Prevention (CDC), 2014, Food and Drug Authority (FDA), 2001, Milstien, 2004). Three phases of clinical trials are required to determine tested vaccine’s effectiveness (immunogenicity) and safety (including adverse events) (Centers for Disease Control and Prevention (CDC), 2014, Food and Drug Authority (FDA), 2001, Milstien, 2004, World Health Organization (WHO), 2014). The United States Food and Drug Administration (USFDA) requires this premarketing evaluation process before granting any vaccine licensure (Centers for Disease Control and Prevention (CDC), 2014). Such evaluations effectively detect most common AEFIs of tested vaccines (Centers for Disease Control and Prevention (CDC), 2014, Ren et al., 2019). However, some AEFIs cannot be detected easily, especially rare ones, either due to the sample size or duration of premarketing trials (Ren et al., 2019).

The Vaccine Adverse Event Reporting System (VAERS) is an early warning system that USFDA and Centers for Disease Control and Prevention (CDC) jointly established in 1990. The main purpose of this system is to collect postmarketing AEFIs reports (VAERS, 1990).In addition, reports in this system can be used to determine if these events are causally related to the vaccination following WHO guideline (World Health Organization, 2019a, World Health Organization, 2019b). This guideline includes a four-step investigation and algorithmic modeling that confirm or deny the causal link between an AEFI and vaccination as following: the temporal association between vaccination and AEFI, a reasonable window between vaccination and AEFI, assessment of other confounders which could cause this adverse event, and literature validation of similar causality(Bellavite et al., 2020, Government of Canada, 2019, Lee et al., 2020, World Health Organization, 2019a, World Health Organization, 2019b). Moreover, VAERS received more attention following coronavirus disease 2019 (COVID-19). Majority of COVID-19 vaccines were approved under emergency use authorization (EUA), which require health care providers to record certain AEFIs in VAERS, including death (FDA, CBER, 2021, Food and Drug Authority (FDA), 2020).

Since VAERS establishment, no study has been conducted to evaluate it comprehensively. Therefore, we aimed to provide a descriptive analysis of VAERS data from its inception through 2021, focusing on pattern analysis, reported AEFIs, most frequently reported symptoms, and adverse events of special interest (AESIs) associated with COVID-19. Last, we aimed to calculate serious AEFIs survival probability.

## 2 Methods

### 2.1 Study design

This was a retrospective observational study using VAERS from the first available report on 02-07-1990 through 12-11-2021.

### 2.2 Data sources

This study uses VAERS data, which is reported by individual health care practitioners or the public when experiences an AEFI without a confirmed cause. Data available on the website is formatted as ready-to-download comma-separated value (CSV) files, which can be imported to CDC WONDER online search tool. VAERS data are available as domestic and nondomestic data. More than 99 % of nondomestic data is reported by vaccine manufacturers. Table 1 shows variables that we used in this study from VAERS. Reports includes two main classifications of the reported AEFIs. Serious AEFIs which are the symptoms that leads to one of the following: death, ER or doctor visit, hospitalization, lethal threat, disability, or nonserious AEFIs (VAERS, 1990). The code of federal regulations (CFR) defines a lethal threat as any adverse event or condition that puts the subject in immediate danger of death. Furthermore, it defines disability as a long-term or significant impairment in the subject's ability to perform normal living functions (FDA, 2018). Moreover, an AESI is a condition or event that occurs in certain people after vaccination and has the potential to be causally linked to a vaccine (FDA, CBER, 2020, Lai et al., 2022, Li et al., 2021a). AESIs we included in this study are anaphylaxis, appendicitis, bell’s palsy, deep vein thrombosis (DVT), disseminated intravascular coagulation, encephalomyelitis, Guillain-Barré syndrome, immune thrombocytopenia, myocardial infarction, myocarditis or pericarditis, narcolepsy, pulmonary embolism, non-hemorrhagic or hemorrhagic stroke, and transverse myelitis (FDA, CBER, 2020, Lai et al., 2022, Li et al., 2021a).

**Table 1:** VAERS data variables names and descriptions.

Variable	Description
**VAERS_ID**	VAERS identification number which is available in the data, symptoms, and vaccines files.
**RECVDATE**	Receiving data of the report, which has been converted to year.
**AGE_YRS**	Age in years.
**SEX**	Reported gender.
**DIED**	Patient died following vaccination.
**L_THREAT**	Patient has life-threatening illness any time following vaccination.
**ER_VISIT**	Patient visits ER any time following vaccination.
**HOSPITAL**	Patient been hospitalized any time following vaccination.
**HOSPDAYS**	Number of hospitalization days.
**DISABLE**	Patient has disability any time following vaccination.
**VAX_TYPE**	Administered vaccine type
**VAX_NAME**	Vaccination name
**SYMPTOM**	Adverse event as per MedDRA*

* MedDRA is an international medical terminology.

### 2.3 Data reprocesses

VAERS data is a multidimensional data, meaning one report may contain several symptoms or vaccines. First, for domestic data, we merged symptoms and vaccine files based on VAERS_ID. Then we separated reports containing multiple vaccines—because no clear information is available about which vaccine triggered those events—in a way that each vaccine reported in them presents the same event information. We followed same steps with nondomestic files. Reprocessing completed using R studio (version 1.4.1717).

### 2.4 Data analysis

We first described patterns of reports over the years using charts by calculating the number of reports per year. We then calculated a comparison of reports’ statistics per year as follows:

(1) $$Percentage\;of\;total\;reports\;in\;a\;year\;=\;\frac{Number\;of\;reports\;for\;each\;year}{Total\;number\;of\;reports\;over\;31\;years}\times 100$$

Furthermore, we compared incidents’ rates (death, ER visits, etc.) in a year as follows:

(2) $$Percentage\;of\;total\;incidents\;in\;a\;year\;=\;\frac{Number\;of\;incidents\;per\;year}{Number\;of\;reports\;per\;year}\times 100$$

In addition, we calculated yearly statistics for each vaccine, and we then described top vaccines’ patterns:

(3) $$Percentage\;of\;reported\;vaccine\;=\;\;\frac{Number\;of\;reports\;containing\;vaccine}{Total\;number\;of\;reports\;over\;31\;years\;}\times 100$$

Moreover, we calculated the incidents’ statistics (death, ER visits, etc.) for each vaccine, and we reported top vaccines.

(4) $$Incident\;rate\;per\;vaccine\;=\;\frac{Number\;of\;incidents\;per\;vaccine}{Total\;number\;of\;incedents}\times 100$$

We made these calculations and visualization using Excel for Microsoft 365. We excluded 2019 coronavirus disease (COVID-19) vaccines from the charts of top vaccines’ patterns due to their recent development.

Moreover, we performed a survival analysis, were individuals followed up from the vaccination date up to the onset of AEFIs, using survival package in R studio (Kartsonaki, 2016). Based on FDA protocol recommendation, we restrict follow up to 365 days (FDA, CBER, 2020, Kochhar et al., 2019). All data in VAERS are for confirmed events, because of that we used non serious AEFIs – instead of no event - as a comparator to serious AEFIs to calculate the probability of survival (Kartsonaki, 2016).

Last, we calculated distribution of AESIs based on age and gender among all vaccines, and their distribution to COVID vaccines.

## 3 Results

### 3.1 Domestic data

There were 1 396 311 reports from 1990 through 2021, however, we excluded 31 reports because they were containing wrong values in majority of variables (e.g: ambiguous text instead of Y or N). We found that 198 025 (14.18 %) have missing age, 867 548 (62.13 %) have missing reporting date, 1 341 680 (96.09 %) have missing hospitalization date, 143 123 (10.25 %) have missing vaccination date, 191 710 (13.72 %), have missing onset date, 229 501 (16.43 %) have missing number of hospitalization days, and 15 357 (1.09 %) have unknown vaccine type. We included the 1 396 280 reports in all this study analysis, except for the survival analysis were only reports with complete vaccination and onset date were included. (Supplementary Fig. 1).

Reports included 228 vaccines. Most reports were recorded in 2021 (n = 677 514, 48.52 %), followed by 2020 (n = 50 205, 3.60 %), 2018 (n = 49 137, 3.52 %), 2019 (n = 48 443, 3.47 %), and 2016 (n = 45 706, 3.27 %). Majority of the samples were from female subjects (60.90 %), and the subjects’ average age was 27.8 years (max = 119, min = <1 month).

COVID-19 vaccines were the top 3 reported vaccines—Moderna (19.81 %), Pfizer-Biontech (19.45 %), and Janssen (4.46 %)—followed by Zoster (Shingrix; 4.10 %), seasonal influenza (Fluzone; 2.88 %), Pneumo (Pneumovax; 2.87 %), Zoster live (Zostavax; 2.41 %), HPV (Gardasil; 2.38 %), Varicella (Varivax; 2.08 %), and measles-mumps-rubella (MMR II; 1.96 %).

#### 3.1.1 Reported deaths

The total number of reported deaths is 13 926 (1.00 %), with most deaths reported in 1990 (3.62 %), followed by 1991 (2.62 %), and 1994 (2.19 %). (Supplementary Fig. 2).

Top vaccines in terms of reported deaths are Pfizer-Biontech (27.98 %), Moderna (24.01 %), Janssen (7.16 %), seasonal influenza, no brand (3.24 %), and Fluzone (2.91 %). (Supplementary Fig. 3).

#### 3.1.2 Reported ER or doctor office visits

The total number of reported ER visits is 186 645 (13.37 %), with top ER visits occurring in 2007 (47.31 %). (Supplementary Fig. 5)

Fluzone (7.22 %) is the top vaccine in terms of reported ER visits, followed by Gardasil (6.00 %), Pneumovax (4.98 %), Varivax (4.05 %), and MMRII (4.03 %). (Supplementary Fig. 6).

#### 3.1.3 Reported hospitalizations

The number of reported hospitalizations is 81 538 (5.84 %), and the largest hospitalization rate occurred in 1990 (12.13 %). (Supplementary Fig. 8).

The highest average of hospitalization days (mean = 12.7 days) occurred in 2021, compared to an average of hospitalization days of 6.3 over the past 30 years.

COVID vaccines are the top vaccines in terms of hospitalization—Pfizer-Biontech (28.09 %), Moderna (20.34 %), and Janssen (6.62 %)—followed by seasonal influenza, no brand (2.31 %), Fluzone (2.28 %), and Gardasil (2.07 %). (Supplementary Fig. 9).

#### 3.1.4 Reported lethal threats

The number of reported lethal threats is 19 872 (1.42 %), with top reported lethal threats occurring in 1999 (2.56 %), followed by 2.03 % in 1995 and 2.00 % in 1994. (Supplementary Fig. 11).

COVID vaccines are the top vaccines in terms of lethal threats—Pfizer-Biontech (22.35 %), Moderna (18.23 %), and Janssen (6.04 %)—followed by Fluzone (4.13 %), Gardasil (3.76 %), and seasonal influenza, no brand (2.21 %). (Supplementary Fig. 12).

#### 3.1.5 Reported disabilities

The total number of reported disabilities is 22 639 (1.4 %), with highest reported disabilities occurring in 1990 (3.95 %), followed by 2002 (2.87 %) and 2001 (2.81 %). (Supplementary Fig. 14).

Pfizer-Biontech is the top vaccine in terms of reported disabilities (20.04 %), followed by Moderna (15.55 %), Gardasil (5.35 %), Janssen (4.58 %), and Zostavax (4.29 %). (Supplementary Fig. 15).

### 3.2 Nondomestic data

There were 346 210 nondomestic reports from 1990 through 2021. We found 54 053 (15.61 %) have missing vaccination date, 53 312 (15.39 %), have missing onset date, and 3 180 (0.92 %) have unknown vaccine type. All these reports were included in this study analysis, except for the survival analysis were only reports with complete vaccination and onset date were included.

Reports included 214 vaccines. 2021 had the most reports (n = 243 500, 70.33 %), followed by 2019 (n = 9,569, 2.76 %), 2018 (n = 8,673, 2.512 %), 2020 (n = 8,111, 2.34 %), and 2016 (n = 7,991, 2.31 %). Most of the sample (61.15 %) came from female subjects, and the average age was 20.94 years. (Supplementary Fig. 1).

The top vaccine in terms of nondomestic reports is Pfizer-Biontech (57.64 %), followed by Moderna (9.04 %), Gardasil (2.57 %), Janssen (2.48 %), and seasonal influenza, no brand name (1.76 %).

#### 3.2.1 Reported deaths

The total number of reported deaths is 14 125 (4.08 %). Most deaths occurred in 1991 (10.17 %), followed by 1993 (9.32 %), 1994 (8.70 %), 1990 (8.16 %), and 1992 (8.00 %). (Supplementary Fig. 2).

The top vaccine in terms of reported deaths is Pfizer-Biontech (61.27 %), followed by Moderna (6.77 %), Janssen (3.88 %), seasonal influenza, no brand name (2.43 %), and Pneumovax (1.67 %). (Supplementary Fig. 4).

#### 3.2.2 Reported ER or doctor office visits

The total number of reported ER or doctor office visits is 7,868 (2.27 %), with most ER or doctor visits occurring in 2015 (24.50 %), followed by 2014 (19.83 %), 2013 (18.07 %), 2016 (17.72 %), and 2000 (11.42 %). (Supplementary Fig. 5).

The top vaccine in terms of reported ER or doctor visits is Gardasil (21.59 %), followed by Pneumovax (9.13 %), Infanrix Hexa (5.31 %), seasonal influenza, no brand name (4.49 %), and Rotateq (4.31 %). (Supplementary Fig. 7).

#### 3.2.3 Reported hospitalization

The total number of reported hospitalizations is 96 166 (27.78 %) with hospitalization occurring mostly in 1994 (76.40 %), followed by 1993 (72.67 %), 1997 (68.44 %), 1995 (66.54 %), and 1996 (65.07 %). (Supplementary Fig. 8).

The average number of hospitalization days in nondomestic reports was 6.10, with the longest average hospital stay occurring in 1992 (mean = 19.67).

Pfizer-Biontech is the top vaccine in terms of reported hospitalizations (45.80 %), followed by Moderna (8.12 %), Gardasil (3.36 %), Pneumovax (2.46 %), and Infanrix Hexa (2.32 %). (Supplementary Fig. 10).

#### 3.2.4 Reported lethal threats

The number of reported lethal threats is 15 151 (4.38 %), with top reported lethal threats occurring in 1992 (17.60 %), followed by 1991 (10.17 %), 1994 (9.94 %), 1995 (6.99 %), and 2000 (6.85 %). (Supplementary Fig. 11).

COVID-19 vaccines were the top vaccines in terms of reported lethal threats—Pfizer-Biontech (60.89 %), Moderna (9.16 %), and Janssen (2.83 %)—followed by Infanrix Hexa (1.89 %) and seasonal influenza, no brand name (1.80 %). (Supplementary Fig. 13).

#### 3.2.5 Reported disabilities

The total number of reported disabilities is 27 545 (7.96 %), with top reported disabilities occurring in 1990 (22.45 %), followed by 2001 (18.21 %), 2002 (16.71 %), 1999 (15.76 %), and 2003 (14.59 %). (Supplementary Fig. 14).

Pfizer-Biontech is the top vaccine in terms of nondomestic reported disabilities (59.52 %), followed by Moderna (10.36 %), Gardasil (4.76 %), Engerix-B (3.42 %), and Janssen (2.70 %). (Supplementary Fig. 16).

### 3.3 Top reported symptoms

The total number of domestic reported symptoms is 3 449 887, while for nondomestic reported symptoms is 2 159 135. Table 2 lists top vaccines in terms of reported symptoms. In addition, Table 3 presents the top reported AEFIs and their associated vaccines.

**Table 2:** Top 10 vaccines in reported symptoms.

Domestic		Non-Domestic	
Vaccine	no.(%)	Vaccines	no.(%)
COVID19 (Moderna)	880,006 (25.51)	COVID19 (COVID19 (PFIZER-BIONTECH))	1,097,734 (50.84)
COVID19 (Pfizer-Biontech)	864,213 (25.05)	COVID19 (COVID19 (**MODERNA))**	136,895 (6.34)
COVID19 (Janssen)	198,136 (5.74)	HPV (GARDASIL)	69,362 (3.21)
Zoster (Shingrix)	182,011 (5.28)	PNEUMO (PREVNAR13)	60,813 (2.82)
Influenza (Seasonal) **(Fluzone)**	123,257 (3.57)	ROTAVIRUS (ROTARIX)	38,758 (1.8)
Pneumo (Pneumovax)	117,097 (3.39)	INFLUENZA (SEASONAL) (NO **BRAND NAME)**	38,702 (1.79)
HPV (Gardasil)	93,746 (2.72)	HEP B (ENGERIX-B**)**	38,499 (1.78)
Zoster live (Zostavax)	92,977 (2.7)	PNEUMO (PNEUMOVAX)	32,520 (1.51)
Influenza (Seasonal) (no brand name)	63,899 (1.85)	COVID19 (COVID19 (**JANSSEN))**	32,017 (1.48)
**Measles + Mumps + Rubella (MMR II)**	62,228 (1.8)	**HPV (CERVARIX)**	30,053 (1.39)

**Table 3:** Top reported AEFI with top related vaccines.

1990–2000 top 5 adverse events				2001–2010 top 5 adverse events			
Domestic		Non-domestic		Domestic		Non-domestic	
Pyrexia	30,164 (8.41 %)	Pyrexia	1439 (5.27 %)	Pyrexia	34,544 (4.59 %)	Pyrexia	9241 (4.28 %)
DTP (NO BRAND NAME)	18.27 %	MEASLES + MUMPS + RUBELLA (MMR II)	14.18 %	INFLUENZA (SEASONAL) (FLUZONE)	7.71 %	PNEUMO (PREVNAR)	10.89 %
DTP (TRI-IMMUNOL)	13.27 %	HEP B (ENGERIX-B)	12.37 %	DTAP (INFANRIX)	7.36 %	POLIO VIRUS, INACT. (NO BRAND NAME)	7.94 %
DTP + HIB (TETRAMUNE)	8.21 %	DTP (NO BRAND NAME)	6.67 %	MEASLES + MUMPS + RUBELLA (MMR II)	5.97 %	DTAP (INFANRIX)	7.72 %
**Injection site hypersensitivity**	**15,379 (4.29 %)**	**Infection**	**1439 (2.44 %)**	**Injection site erythema**	**33,272 (4.42 %)**	**Vomiting**	**3260 (1.51 %)**
TD ADSORBED (NO BRAND NAME)	15.44 %	HEP B (ENGERIX-B)	20.21 %	DTAP (INFANRIX)	13.54 %	PNEUMO (PREVNAR)	9.54 %
DTP (NO BRAND NAME)	10.22 %	MEASLES + MUMPS + RUBELLA (MMR II)	10.03 %	PNEUMO (PNEUMOVAX)	10.06 %	ROTAVIRUS (ROTARIX)	9.26 %
DTAP (TRIPEDIA)	7.02 %	HEP B (RECOMBIVAX HB)	7.49 %	DTAP (TRIPEDIA)	9.00 %	POLIO VIRUS, INACT. (NO BRAND NAME)	6.69 %
**Rash**	**12,034 (3.36 %)**	**Laboratory test abnormal**	**655 (2.4 %)**	**Erythema**	**20,638 (2.74 %)**	**Body temperature increased**	**3185 (1.47 %)**
MEASLES + MUMPS + RUBELLA (MMR II)	14.21 %	HEP B (ENGERIX-B)	38.02 %	DTAP (INFANRIX)	10.01 %	PNEUMO (PREVNAR)	16.99 %
VARICELLA (VARIVAX)	10.55 %	HEP B (RECOMBIVAX HB)	9.16 %	PNEUMO (PNEUMOVAX)	9.68 %	POLIO VIRUS, INACT. (NO BRAND NAME)	9.92 %
HEP B (ENGERIX-B)	10.52 %	MEASLES + MUMPS + RUBELLA (MMR II)	8.70 %	DTAP (DAPTACEL)	8.71 %	DTAP (INFANRIX)	8.98 %
**Injection site oedema**	**11,268 (3.14 %)**	**Convulsion**	**513 (1.88 %)**	**Injection site swelling**	**19,202 (2.55 %)**	**Crying**	**3051 (1.41 %)**
TD ADSORBED (NO BRAND NAME)	16.02 %	MEASLES + MUMPS + RUBELLA (MMR II)	11.89 %	PNEUMO (PNEUMOVAX)	11.68 %	PNEUMO (PREVNAR)	13.67 %
DTP (NO BRAND NAME)	11.73 %	HEP B (ENGERIX-B)	11.11 %	DTAP (INFANRIX)	11.11 %	POLIO VIRUS, INACT. (NO BRAND NAME)	10.62 %
DTAP (TRIPEDIA)	7.26 %	HEP B (RECOMBIVAX HB)	8.19 %	DTAP (DAPTACEL)	8.04 %	HIB (ACTHIB)	9.73 %
**Vasodilatation**	**10,339 (2.88 %)**	**Vomiting**	**407 (1.49 %)**	**Injection site pain**	**17,325 (2.3 %)**	**Pallor**	**2958 (1.37 %)**
TD ADSORBED (NO BRAND NAME)	14.62 %	HEP B (ENGERIX-B)	17.44 %	PNEUMO (PNEUMOVAX)	14.46 %	PNEUMO (PREVNAR)	13.56 %
DTP (NO BRAND NAME)	8.78 %	MEASLES + MUMPS + RUBELLA (MMR II)	10.32 %	INFLUENZA (SEASONAL) (FLUZONE)	10.05 %	POLIO VIRUS, INACT. (NO BRAND NAME)	12.04 %
DTAP (TRIPEDIA)	6.79 %	DTP (NO BRAND NAME)	6.88 %	HPV (GARDASIL)	6.49 %	DTAP (INFANRIX)	10.92 %

**2011**–**2020 top 5 adverse events**				**2021 top 5 adverse events**			

**Domestic**		**Non-domestic**		**Domestic**		**Non-domestic**	

**Pyrexia**	**45,536 (3.33 %)**	**Pyrexia**	**23,397 (3.8 %)**	**Headache**	**95,058 (4.84 %)**	**SARS-CoV-2 test**	**59,666 (4.59 %)**

ZOSTER (SHINGRIX)	20.76 %	PNEUMO (PREVNAR13)	10.83 %	COVID19 (COVID19 (MODERNA))	43.75 %	COVID19 (PFIZER-BIONTECH)	88.86 %
PNEUMO (PNEUMOVAX)	5.23 %	MENINGOCOCCAL B (BEXSERO)	5.88 %	COVID19 (COVID19 (PFIZER-BIONTECH))	40.77 %	COVID19 (MODERNA)	9.24 %
INFLUENZA (SEASONAL) (FLUZONE HIGH-DOSE)	3.97 %	PNEUMO (PNEUMOVAX)	5.58 %	COVID19 (COVID19 (JANSSEN))	12.98 %	COVID19 (JANSSEN)	1.50 %
**Injection site pain**	**38,934 (2.85 %)**	**Vomiting**	**8650 (1.40 %)**	**Pyrexia**	**82,643 (4.21 %)**	**Headache**	**41,312 (3.18 %)**
ZOSTER (SHINGRIX)	18.99 %	ROTAVIRUS (ROTARIX)	13.03 %	COVID19 (COVID19 (MODERNA))	46.77 %	COVID19 (PFIZER-BIONTECH)	80.10 %
PNEUMO (PNEUMOVAX)	10.58 %	PNEUMO (PREVNAR13)	9.23 %	COVID19 (COVID19 (PFIZER-BIONTECH))	37.11 %	COVID19 (MODERNA)	14.96 %
ZOSTER LIVE (ZOSTAVAX)	5.67 %	ROTAVIRUS (ROTATEQ)	6.10 %	COVID19 (COVID19 (JANSSEN))	12.79 %	COVID19 (JANSSEN)	3.83 %
**Injection site erythema**	**37,276 (2.73 %)**	**Headache**	**7266 (1.18 %)**	**Fatigue**	**73,491 (3.74 %)**	**Fatigue**	**33,405 (2.57 %)**
ZOSTER LIVE (ZOSTAVAX)	14.31 %	HPV (GARDASIL)	27.00 %	COVID19 (COVID19 (MODERNA))	45.07 %	COVID19 (PFIZER-BIONTECH)	81.10 %
PNEUMO (PNEUMOVAX)	10.26 %	HPV (CERVARIX)	10.38 %	COVID19 (COVID19 (PFIZER-BIONTECH))	41.77 %	COVID19 (MODERNA)	14.64 %
ZOSTER (SHINGRIX)	6.22 %	INFLUENZA (SEASONAL) (NO BRAND NAME)	5.78 %	COVID19 (COVID19 (JANSSEN))	10.76 %	COVID19 (JANSSEN)	3.23 %
**Incorrect product storage**	**29,322 (2.15 %)**	**Crying**	**7149 (1.16 %)**	**Pain**	**71,897 (3.66 %)**	**Pyrexia**	**33,118 (2.55 %)**
VARICELLA (VARIVAX)	16.57 %	PNEUMO (PREVNAR13)	13.50 %	COVID19 (COVID19 (MODERNA))	43.86 %	COVID19 (PFIZER-BIONTECH)	71.93 %
ROTAVIRUS (ROTATEQ)	12.27 %	ROTAVIRUS (ROTARIX)	9.22 %	COVID19 (COVID19 (PFIZER-BIONTECH))	39.97 %	COVID19 (MODERNA)	20.01 %
MEASLES + MUMPS + RUBELLA + VARICELLA (PROQUAD)	8.79 %	HIB (ACTHIB)	8.71 %	COVID19 (COVID19 (JANSSEN))	12.17 %	COVID19 (JANSSEN)	4.19 %
**Injection site swelling**	**27,930 (2.04 %)**	**Diarrhoea**	**5913 (0.96 %)**	**Nausea**	**55,723 (2.84 %)**	**Nausea**	**23,607 (1.82 %)**
ZOSTER LIVE (ZOSTAVAX)	12.09 %	ROTAVIRUS (ROTARIX)	18.03 %	COVID19 (COVID19 (MODERNA))	43.68 %	COVID19 (PFIZER-BIONTECH)	79.76 %
PNEUMO (PNEUMOVAX)	12.00 %	PNEUMO (PREVNAR13)	9.25 %	COVID19 (COVID19 (PFIZER-BIONTECH))	41.94 %	COVID19 (MODERNA)	15.73 %
ZOSTER (SHINGRIX)	5.66 %	ROTAVIRUS (ROTATEQ)	8.30 %	COVID19 (COVID19 (JANSSEN))	11.75 %	COVID19 (JANSSEN)	3.34 %

### 3.4 Survival analysis results

Reports with complete vaccination and onset data are 1 121 698, and 262 723, for domestic and nondomestic data, respectively. Probability of having non serious AEFIs has its highest value at the vaccination’s day, with 93 % probability of having non serious AEFIs in domestic data, and 85.91 % in nondomestic data. It continuously decreased after that to its minimal 10.40 % and 6.08 % at the end of the following up window. However, for serious AEFIs they have a proportional relation to time, where they have their lowest values in the vaccination’s day, ranging from 0.09 % to 5.18 % for domestic data and from 0.39 % to 8.00 % for nondomestic data. For domestic data ER or Doctor visit was the highest serious AEFIs by 33.84 %, and for nondomestic data hospitalization was the highest by 54.10 % at the end of the follow up window. Table 4 contains survival analysis of both domestic and nondomestic data. Cox regression results of both domestic and nondomestic data shows that males have more probability to have AEFIs than females (HR: 1.15, 95 % confidence interval (95 % CI): 1.14–1.16) in domestic, and (HR: 1.23, 95 % CI: 1.22–1.25) in nondomestic. While for age group, in domestic data age group ≤ 5 years have a higher probability of having AEFIs compared to other groups. However, in nondomestic data age group >84 years have a higher probability of having AEFIs compared to other groups (HR: 2.15, 95 % CI: 2.07–2.23). Table 5 presents hazard ration with 95 % CI for domestic and nondomestic data.

**Table 4:** Survival analysis of domestic and nondomestic data.

Domestic Data Survival Analysis												
Days	n. risk	n. event	P (n. AEFIs)	st.er	L 95 % CI	U 95 % CI	P (D)	P (ED)	P (H)	P (LT)	P (DA)	P (m. AEFIs)
0	1,112,242	78,028	0.93	0.00	0.93	0.93	0.00	0.05	0.01	0.00	0.00	0.01
1	597,977	49,420	0.85	0.00	0.85	0.85	0.00	0.11	0.01	0.00	0.00	0.02
2	354,436	19,049	0.81	0.00	0.81	0.81	0.00	0.14	0.02	0.00	0.01	0.02
3	281,684	9043	0.78	0.00	0.78	0.78	0.01	0.15	0.02	0.00	0.01	0.03
4	248,551	5601	0.76	0.00	0.76	0.77	0.01	0.16	0.03	0.00	0.01	0.03
5	228,377	4565	0.75	0.00	0.75	0.75	0.01	0.17	0.03	0.00	0.01	0.03
6	212,285	3840	0.74	0.00	0.73	0.74	0.01	0.18	0.03	0.00	0.01	0.04
7	197,700	5425	0.72	0.00	0.71	0.72	0.01	0.19	0.04	0.00	0.01	0.04
30	60,908	33,564	0.52	0.00	0.52	0.52	0.02	0.26	0.08	0.01	0.02	0.09
60	37,745	7723	0.44	0.00	0.44	0.44	0.02	0.28	0.11	0.01	0.03	0.11
90	29,066	3299	0.40	0.00	0.40	0.40	0.03	0.29	0.12	0.01	0.03	0.12
100	26,608	895	0.39	0.00	0.39	0.39	0.03	0.29	0.13	0.01	0.03	0.13
200	8884	8045	0.23	0.00	0.23	0.24	0.04	0.31	0.22	0.01	0.04	0.16
300	1292	3378	0.10	0.00	0.10	0.11	0.04	0.34	0.29	0.01	0.04	0.17
**Non-Domestic Data Survival Analysis**												

**days**	**n.** **risk**	**n.** **event**	**P** **(n. AEFIs)**	st.er	**L** **95 % CI**	**U** **95 % CI**	**P** **(D)**	**P** **(ED)**	**P** **(H)**	**P** **(LT)**	**P** **(DA)**	**P** **(m. AEFIs)**

0	260,378	36,695	0.86	0.00	0.86	0.86	0.01	0.00	0.08	0.01	0.03	0.02
1	155,488	16,485	0.77	0.00	0.77	0.77	0.01	0.01	0.13	0.01	0.05	0.03
2	112,050	7030	0.72	0.00	0.72	0.72	0.02	0.01	0.15	0.01	0.06	0.03
3	96,014	4934	0.68	0.00	0.68	0.69	0.02	0.01	0.18	0.02	0.06	0.04
4	85,967	3416	0.66	0.00	0.65	0.66	0.02	0.01	0.19	0.02	0.06	0.04
5	79,038	2818	0.63	0.00	0.63	0.63	0.02	0.01	0.21	0.02	0.07	0.05
6	73,505	2319	0.61	0.00	0.61	0.61	0.03	0.01	0.22	0.02	0.07	0.05
7	68,875	2691	0.59	0.00	0.59	0.59	0.03	0.01	0.23	0.02	0.07	0.05
30	26,492	19,339	0.37	0.00	0.37	0.37	0.04	0.01	0.36	0.03	0.09	0.09
60	13,739	5328	0.28	0.00	0.28	0.29	0.05	0.01	0.42	0.03	0.10	0.11
90	8299	1996	0.23	0.00	0.23	0.24	0.05	0.01	0.44	0.03	0.11	0.12
100	7181	419	0.22	0.00	0.22	0.22	0.05	0.01	0.45	0.03	0.11	0.12
200	1925	2058	0.13	0.00	0.13	0.14	0.06	0.01	0.50	0.04	0.12	0.14
300	417	719	0.06	0.00	0.06	0.07	0.06	0.02	0.54	0.04	0.13	0.15

**Days.** days from vaccination to AEFIs (serious or nonserious), **n.risk.** number of risks**, n.event.** number of events, **P(n.AEFIs).** probability of nonserious AEFIs, st.er. standard error, **L.95%.CI.** lower 95% confidence interval, **U.95%.CI.** upper 95% confidence interval, **P(D).** probability of death, **P(ED).** probability of ER or doctor visit, **P(H).** probability of hospitalization, **P(LT).** probability of lethal threat, **P(DA).** probability of disability, **P(m.AEFIs).** probability of multibed AEFIs.

**Table 5:** **COX** regression results of both domestic and nondomestic data.

	Domestic				Nondomestic		
Characteristic	HR	95 % CI	p-value		HR	95 % CI	p-value
**Gender (Female is the control)**							
Male	1.15	1.14, 1.16	<0.001		1.23	1.22, 1.25	<0.001
Unknown	0.31	0.29, 0.32	<0.001		1.43	1.38, 1.48	<0.001
**Age (≤5 years is the control)**							
6–17 years	0.8	0.78, 0.81	<0.001		1.43	1.39, 1.47	<0.001
18–34 years	0.41	0.41, 0.42	<0.001		1.24	1.21, 1.27	<0.001
35–54 years	0.31	0.31, 0.32	<0.001		1.32	1.29, 1.34	<0.001
55–64 years	0.31	0.31, 0.32	<0.001		1.48	1.44, 1.53	<0.001
65–74 years	0.34	0.33, 0.34	<0.001		1.52	1.48, 1.56	<0.001
75–84 years	0.37	0.37, 0.38	<0.001		1.73	1.68, 1.78	<0.001
≥ 85 years	0.45	0.44, 0.46	<0.001		2.15	2.07, 2.23	<0.002
**HR = Hazard Ration, CI = Confidence Interval**							

### 3.5 Adverse event of special interest (AESIs)

Out of 3 449 887 domestic reported symptoms 17 611 (0.51 %) were AESIs, 9 665 (54.88 %) were associated with COVID-19 vaccines. While for nondomestic reported symptoms out of 2 159 135 there were 35 810 (1.66 %) AESIs, 31 511 (87.99 %) were associated with COVID-19 vaccines. Table 6 shows distribution of AESIs associated with COVID-19 stratified by gender and age.

**Table 6:** AESIs -associated with COVID-19 vaccines- distribution based on age and gender.

Outcome by Gender	1–5 years		6–17 years		18–34 years		35–54 years		55–64 years		65–74 years		75–84 years		≥85 years		Total	
	Domestic	Non-Domestic	Domestic	Non-Domestic	Domestic	Non-Domestic	Domestic	Non-Domestic	Domestic	Non-Domestic	Domestic	Non-Domestic	Domestic	Non-Domestic	Domestic	Non-Domestic	Domestic	Non-Domestic
	no.(%)	no.(%)	no.(%)	no.(%)	no.(%)	no.(%)	no.(%)	no.(%)	no.(%)	no.(%)	no.(%)	no.(%)	no.(%)	no.(%)	no.(%)	no.(%)	no.(%)	no.(%)
**Anaphylaxis**	**8 (2.53)**	**996 (5.62)**	**4 (0.69)**	**115 (29.19)**	**43 (2.47)**	**1446 (39.76)**	**82 (3.03)**	**2136 (47.42)**	**29 (1.79)**	**544 (31.21)**	**10 (0.63)**	**278 (18.98)**	**2 (0.26)**	**147 (11.15)**	**1 (0.31)**	**87 (12.17)**	**179 (1.85)**	**5749 (18.24)**
F	8 (100)	806 (80.92)	4 (100))	66 (57.39)	38 (88.37)	1073 (74.2)	68 (82.93)	1817 (85.07)	24 (82.76)	463 (85.11)	7 (70)	219 (78.78)	0 (0)	111 (75.51)	1 (100))	71 (81.61)	150 (83.8)	4626 (80.47)
M	0 (0)	190 (19.08)	0 (0)	49 (42.61)	5 (11.63)	373 (25.8)	14 (17.07)	319 (14.93)	5 (17.24)	81 (14.89)	3 (30)	59 (21.22)	2 (100))	36 (24.49)	0 (0)	16 (18.39)	29 (16.2)	1123 (19.53)
**Appendicitis**	**9 (2.85)**	**218 (1.23)**	**36 (6.23)**	**1 (0.25)**	**123 (7.05)**	**31 (0.85)**	**219 (8.09)**	**36 (0.8)**	**69 (4.26)**	**7 (0.4)**	**45 (2.81)**	**6 (0.41)**	**10 (1.29)**	**4 (0.3)**	**2 (0.62)**	**1 (0.14)**	**513 (5.31)**	**304 (0.96)**
F	8 (88.89)	113 (51.83)	13 (36.11)	0 (0)	88 (71.54)	22 (70.97)	137 (62.56)	22 (61.11)	51 (73.91)	4 (57.14)	31 (68.89)	4 (66.67)	7 (70)	2 (50)	1 (50)	0 (0)	336 (65.5)	167 (54.93)
M	1 (11.11)	105 (48.17)	23 (63.89)	1 (100))	35 (28.46)	9 (29.03)	82 (37.44)	14 (38.89)	18 (26.09)	3 (42.86)	14 (31.11)	2 (33.33)	3 (30)	2 (50)	1 (50)	1 (100))	177 (34.5)	137 (45.07)
**Bell's palsy**	**14 (4.43)**	**1110 (6.26)**	**4 (0.69)**	**5 (1.27)**	**62 (3.56)**	**110 (3.02)**	**157 (5.8)**	**187 (4.15)**	**57 (3.52)**	**84 (4.82)**	**49 (3.06)**	**51 (3.48)**	**8 (1.03)**	**23 (1.75)**	**4 (1.23)**	**5 (0.7)**	**355 (3.67)**	**1575 (5)**
F	10 (71.43)	617 (55.59)	1 (25)	1 (20)	44 (70.97)	53 (48.18)	93 (59.24)	104 (55.61)	30 (52.63)	44 (52.38)	30 (61.22)	30 (58.82)	3 (37.5)	11 (47.83)	3 (75)	4 (80)	214 (60.28)	864 (54.86)
M	4 (28.57)	493 (44.41)	3 (75)	4 (80)	18 (29.03)	57 (51.82)	64 (40.76)	83 (44.39)	27 (47.37)	40 (47.62)	19 (38.78)	21 (41.18)	5 (62.5)	12 (52.17)	1 (25)	1 (20)	141 (39.72)	711 (45.14)
**Deep vein thrombosis**	**20 (6.33)**	**2658 (14.99)**	**9 (1.56)**	**7 (1.78)**	**97 (5.56)**	**110 (3.02)**	**361 (13.34)**	**355 (7.88)**	**245 (15.11)**	**193 (11.07)**	**228 (14.25)**	**204 (13.92)**	**107 (13.82)**	**228 (17.3)**	**52 (16)**	**75 (10.49)**	**1119 (11.58)**	**3830 (12.15)**
F	8 (40)	1340 (50.41)	5 (55.56)	5 (71.43)	64 (65.98)	71 (64.55)	192 (53.19)	174 (49.01)	107 (43.67)	78 (40.41)	111 (48.68)	88 (43.14)	52 (48.6)	127 (55.7)	31 (59.62)	46 (61.33)	570 (50.94)	1929 (50.37)
M	12 (60)	1318 (49.59)	4 (44.44)	2 (28.57)	33 (34.02)	39 (35.45)	169 (46.81)	181 (50.99)	138 (56.33)	115 (59.59)	117 (51.32)	116 (56.86)	55 (51.4)	101 (44.3)	21 (40.38)	29 (38.67)	549 (49.06)	1901 (49.63)
**Disseminated intravascular coagulation**	**2 (0.63)**	**35 (0.2)**	**1 (0.17)**	**1 (0.25)**	**2 (0.11)**	**4 (0.11)**	**14 (0.52)**	**3 (0.07)**	**7 (0.43)**	**3 (0.17)**	**5 (0.31)**	**8 (0.55)**	**1 (0.13)**	**7 (0.53)**	**1 (0.31)**	**9 (1.26)**	**33 (0.34)**	**70 (0.22)**
F	1 (50)	13 (37.14)	0 (0)	0 (0)	2 (100))	3 (75)	10 (71.43)	3 (100))	3 (42.86)	3 (100))	4 (80)	3 (37.5)	0 (0)	2 (28.57)	0 (0)	5 (55.56)	20 (60.61)	32 (45.71)
M	1 (50)	22 (62.86)	1 (100))	1 (100))	0 (0)	1 (25)	4 (28.57)	0 (0)	4 (57.14)	0 (0)	1 (20)	5 (62.5)	1 (100))	5 (71.43)	1 (100))	4 (44.44)	13 (39.39)	38 (54.29)
**Encephalomyelitis**	**0 (0)**	**51 (0.29)**	**0 (0)**	**2 (0.51)**	**4 (0.23)**	**19 (0.52)**	**3 (0.11)**	**20 (0.44)**	**1 (0.06)**	**7 (0.4)**	**0 (0)**	**6 (0.41)**	**1 (0.13)**	**4 (0.3)**	**0 (0)**	**1 (0.14)**	**9 (0.09)**	**110 (0.35)**
F	0 (0)	35 (68.63)	0 (0)	0 (0)	3 (75)	14 (73.68)	2 (66.67)	15 (75)	1 (100))	4 (57.14)	0 (0)	5 (83.33)	0 (0)	3 (75)	0 (0)	0 (0)	6 (66.67)	76 (69.09)
M	0 (0)	16 (31.37)	0 (0)	2 (100))	1 (25)	5 (26.32)	1 (33.33)	5 (25)	0 (0)	3 (42.86)	0 (0)	1 (16.67)	1 (100))	1 (25)	0 (0)	1 (100))	3 (33.33)	34 (30.91)
**Guillain-Barre syndrome**	**24 (7.59)**	**572 (3.23)**	**16 (2.77)**	**3 (0.76)**	**60 (3.44)**	**59 (1.62)**	**148 (5.47)**	**100 (2.22)**	**114 (7.03)**	**48 (2.75)**	**94 (5.88)**	**52 (3.55)**	**36 (4.65)**	**33 (2.5)**	**5 (1.54)**	**8 (1.12)**	**497 (5.14)**	**875 (2.78)**
F	10 (41.67)	272 (47.55)	6 (37.5)	2 (66.67)	35 (58.33)	32 (54.24)	81 (54.73)	64 (64)	53 (46.49)	15 (31.25)	47 (50)	16 (30.77)	11 (30.56)	14 (42.42)	3 (60)	6 (75)	246 (49.5)	421 (48.11)
M	14 (58.33)	300 (52.45)	10 (62.5)	1 (33.33)	25 (41.67)	27 (45.76)	67 (45.27)	36 (36)	61 (53.51)	33 (68.75)	47 (50)	36 (69.23)	25 (69.44)	19 (57.58)	2 (40)	2 (25)	251 (50.5)	454 (51.89)
**Immune thrombocytopenia**	**10 (3.16)**	**365 (2.06)**	**4 (0.69)**	**4 (1.02)**	**34 (1.95)**	**32 (0.88)**	**49 (1.81)**	**52 (1.15)**	**19 (1.17)**	**32 (1.84)**	**38 (2.38)**	**23 (1.57)**	**16 (2.07)**	**24 (1.82)**	**8 (2.46)**	**15 (2.1)**	**178 (1.84)**	**547 (1.74)**
F	6 (60)	212 (58.08)	3 (75)	0 (0)	19 (55.88)	22 (68.75)	36 (73.47)	32 (61.54)	12 (63.16)	16 (50)	18 (47.37)	10 (43.48)	11 (68.75)	10 (41.67)	3 (37.5)	11 (73.33)	108 (60.67)	313 (57.22)
M	4 (40)	153 (41.92)	1 (25)	4 (100))	15 (44.12)	10 (31.25)	13 (26.53)	20 (38.46)	7 (36.84)	16 (50)	20 (52.63)	13 (56.52)	5 (31.25)	14 (58.33)	5 (62.5)	4 (26.67)	70 (39.33)	234 (42.78)
**Myelitis transverse**	**10 (3.16)**	**81 (0.46)**	**1 (0.17)**	**0 (0)**	**18 (1.03)**	**3 (0.08)**	**41 (1.51)**	**15 (0.33)**	**31 (1.91)**	**5 (0.29)**	**18 (1.13)**	**2 (0.14)**	**12 (1.55)**	**1 (0.08)**	**3 (0.92)**	**0 (0)**	**134 (1.39)**	**107 (0.34)**
F	7 (70)	55 (67.9)	0 (0)	0 (0)	12 (66.67)	1 (33.33)	28 (68.29)	8 (53.33)	15 (48.39)	5 (100))	9 (50)	1 (50)	6 (50)	1 (100))	0 (0)	0 (0)	77 (57.46)	71 (66.36)
M	3 (30)	26 (32.1)	1 (100))	0 (0)	6 (33.33)	2 (66.67)	13 (31.71)	7 (46.67)	16 (51.61)	0 (0)	9 (50)	1 (50)	6 (50)	0 (0)	3 (100))	0 (0)	57 (42.54)	36 (33.64)
**Myocardial infarction**	**60 (18.99)**	**1292 (7.29)**	**4 (0.69)**	**4 (1.02)**	**64 (3.67)**	**39 (1.07)**	**231 (8.53)**	**174 (3.86)**	**222 (13.7)**	**139 (7.97)**	**243 (15.19)**	**156 (10.65)**	**146 (18.86)**	**138 (10.47)**	**75 (23.08)**	**111 (15.52)**	**1045 (10.81)**	**2053 (6.52)**
F	28 (46.67)	487 (37.69)	0 (0)	1 (25)	15 (23.44)	17 (43.59)	103 (44.59)	54 (31.03)	88 (39.64)	37 (26.62)	107 (44.03)	63 (40.38)	61 (41.78)	69 (50)	42 (56)	67 (60.36)	444 (42.49)	795 (38.72)
M	32 (53.33)	805 (62.31)	4 (100))	3 (75)	49 (76.56)	22 (56.41)	128 (55.41)	120 (68.97)	134 (60.36)	102 (73.38)	136 (55.97)	93 (59.62)	85 (58.22)	69 (50)	33 (44)	44 (39.64)	601 (57.51)	1258 (61.28)
**Myocarditis or Pericarditis**	**95 (30.06)**	**4975 (28.05)**	**472 (81.66)**	**240 (60.91)**	**993 (56.94)**	**1584 (43.55)**	**584 (21.57)**	**845 (18.76)**	**222 (13.7)**	**235 (13.48)**	**173 (10.81)**	**149 (10.17)**	**53 (6.85)**	**86 (6.53)**	**13 (4)**	**28 (3.92)**	**2605 (26.95)**	**8142 (25.84)**
F	33 (34.74)	1810 (36.38)	56 (11.86)	27 (11.25)	221 (22.26)	367 (23.17)	256 (43.84)	362 (42.84)	121 (54.5)	108 (45.96)	76 (43.93)	63 (42.28)	21 (39.62)	47 (54.65)	6 (46.15)	18 (64.29)	790 (30.33)	2802 (34.41)
M	62 (65.26)	3165 (63.62)	416 (88.14)	213 (88.75)	772 (77.74)	1217 (76.83)	328 (56.16)	483 (57.16)	101 (45.5)	127 (54.04)	97 (56.07)	86 (57.72)	32 (60.38)	39 (45.35)	7 (53.85)	10 (35.71)	1815 (69.67)	5340 (65.59)
**Narcolepsy**	**4 (1.27)**	**29 (0.16)**	**1 (0.17)**	**1 (0.25)**	**2 (0.11)**	**5 (0.14)**	**12 (0.44)**	**8 (0.18)**	**1 (0.06)**	**1 (0.06)**	**3 (0.19)**	**1 (0.07)**	**1 (0.13)**	**3 (0.23)**	**1 (0.31)**	**1 (0.14)**	**25 (0.26)**	**49 (0.16)**
F	3 (75)	20 (68.97)	0 (0)	0 (0)	2 (100))	5 (100))	10 (83.33)	4 (50)	1 (100))	1 (100))	2 (66.67)	1 (100))	0 (0)	1 (33.33)	1 (100))	1 (100))	19 (76)	33 (67.35)
M	1 (25)	9 (31.03)	1 (100))	1 (100))	0 (0)	0 (0)	2 (16.67)	4 (50)	0 (0)	0 (0)	1 (33.33)	0 (0)	1 (100))	2 (66.67)	0 (0)	0 (0)	6 (24)	16 (32.65)
**Non-haemorrhagic or Haemorrhagic stroke**	**7 (2.22)**	**1880 (10.6)**	**9 (1.56)**	**6 (1.52)**	**40 (2.29)**	**27 (0.74)**	**179 (6.61)**	**186 (4.13)**	**154 (9.5)**	**182 (10.44)**	**194 (12.13)**	**272 (18.57)**	**128 (16.54)**	**344 (26.1)**	**72 (22.15)**	**256 (35.8)**	**783 (8.1)**	**3153 (10.01)**
F	6 (85.71)	956 (50.85)	6 (66.67)	2 (33.33)	20 (50)	18 (66.67)	92 (51.4)	86 (46.24)	80 (51.95)	65 (35.71)	100 (51.55)	122 (44.85)	73 (57.03)	183 (53.2)	47 (65.28)	169 (66.02)	424 (54.15)	1601 (50.78)
M	1 (14.29)	924 (49.15)	3 (33.33)	4 (66.67)	20 (50)	9 (33.33)	87 (48.6)	100 (53.76)	74 (48.05)	117 (64.29)	94 (48.45)	150 (55.15)	55 (42.97)	161 (46.8)	25 (34.72)	87 (33.98)	359 (45.85)	1552 (49.22)
**Pulmonary embolism**	**53 (16.77)**	**3473 (19.58)**	**17 (2.94)**	**5 (1.27)**	**202 (11.58)**	**168 (4.62)**	**627 (23.16)**	**387 (8.59)**	**450 (27.76)**	**263 (15.09)**	**500 (31.25)**	**257 (17.54)**	**253 (32.69)**	**276 (20.94)**	**88 (27.08)**	**118 (16.5)**	**2190 (22.66)**	**4947 (15.7)**
F	28 (52.83)	1769 (50.94)	12 (70.59)	3 (60)	136 (67.33)	104 (61.9)	345 (55.02)	169 (43.67)	205 (45.56)	86 (32.7)	249 (49.8)	112 (43.58)	135 (53.36)	157 (56.88)	51 (57.95)	75 (63.56)	1161 (53.01)	2475 (50.03)
M	25 (47.17)	1704 (49.06)	5 (29.41)	2 (40)	66 (32.67)	64 (38.1)	282 (44.98)	218 (56.33)	245 (54.44)	177 (67.3)	251 (50.2)	145 (56.42)	118 (46.64)	119 (43.12)	37 (42.05)	43 (36.44)	1029 (46.99)	2472 (49.97)
**Grand Total**	**316 (3.27)**	**17,735 (56.28)**	**578 (5.98)**	**394 (1.25)**	**1744 (18.04)**	**3637 (11.54)**	**2707 (28.01)**	**4504 (14.29)**	**1621 (16.77)**	**1743 (5.53)**	**1600 (16.55)**	**1465 (4.65)**	**774 (8.01)**	**1318 (4.18)**	**325 (3.36)**	**715 (2.27)**	**9665** (100)**)**	**31,511** (100)**)**

## 4 Discussion

Vaccination saves 4 to 5 million people from death each year, according to WHO estimates (World Health Organization, 2019a, World Health Organization, 2019b). Moreover, it is the most effective technique for preventing infectious diseases and reducing their associated mortality and morbidity rate (Dubé et al., 2013). However, threatening factors associated with vaccinations, such as AEFIs, could have a negative impact on vaccination rates (Dubé et al., 2013). Here, we describe VAERS data and evaluate their usability. We also examine pandemics’ impact on the reporting patterns.

VAERS provides a wealth of information for analyzing AEFIs and their patterns over time (Ren et al., 2019, Shimabukuro et al., 2015, VAERS, 1990). Such a surveillance system with a large data set can be utilized for event detection and generation to investigate and evaluate vaccine safety, ultimately helping develop regulatory laws and actions to minimize risks associated with these vaccines (Ren et al., 2019, Shimabukuro et al., 2015, Varricchio et al., 2004). Descriptive and pattern analysis of raw VAERS data combined with data visualization showed several intriguing patterns over the previous 31 years. This study’s outcomes provide some insights into and can guide further research on certain vaccines and AEFIs.

COVID-19 vaccine was released under EUA, which requires reporting of any AEFI occurrence (FDA, CBER, 2021, Food and Drug Authority (FDA), 2020). VAERS data shows a huge increase in reporting in 2021, accounting for 48.52 % of domestic and 70.33 % of nondomestic reports over the past 31 years. Furthermore, COVID-19 vaccines were the top reported vaccines, accounting for 43.72 % of domestic and 69.16 % of nondomestic total reported vaccines.

A major component of VAERS design is the identification of serious AEFIs, such as death, hospitalization, lethal threat, and permanent disability (Food and Drug Authority (FDA), 2016). The reporting rate of domestic deaths steadily decreased for 30 years until 2021, where it started rising. However, underreporting clearly occurred, with reported deaths representing only 1 % and 4.08 % of domestic and nondomestic data, respectively. The fact that the pneumococcal conjugate vaccine was released in 2000 may explain the increase in nondomestic reported deaths, which also occurred in 2021, when COVID-19 vaccine was released (DeStefano, 2008, FDA, CBER, 2021, Hause et al., 2020, Jefferies et al., 2011, Ryan Gierke et al., 2021). The number of Pnumovax-associated deaths increased in 2000, confirming our suggestion (DeStefano, 2008, Jefferies et al., 2011, Ryan Gierke et al., 2021). Another pattern worth noting is that reported domestic deaths associated with influenza vaccines increased in 2011. We found a similar increase in 2019. Influenza H1N1 was a pandemic disease that struck between 2009 and 2010; as a result, CDC recommended administering influenza vaccines to all persons 6 months or older (Campbell and Grohskopf, 2018, Fiore et al., 2010). In 2019, US health strategy to manage COVID-19 pandemic included influenza vaccination to minimize influenza-related respiratory diseases and optimize health care services for COVID-19 patients (Grohskopf et al., 2021). Furthermore, nondomestic reported deaths show an interesting pattern associated with DTP, whose occurrence sudden increased in 2018, possibly due to an increase in pertussis cases in 2018 and 2019, as CDC reported more than 15 600 cases per year (Centers for Disease Control and Prevention, 2022).

Domestic ER or doctor office visit reports show an upward trend from 1990 to 2007, then show a downward pattern until they flatten. However, for nondomestic ER or doctor office visits, the pattern fluctuated steadily until 2011, when it began to rise, reaching its peak in 2015. Among the vaccines most frequently associated with nondomestic reported ER or doctor visits, HPV vaccine showed a similar pattern. In addition, a similar increase in domestic reported ER or doctor visits was associated with HPV from 2006 to 2008, with a steady decline following. USFDA first approved HPV vaccine in 2006, which may explain the increase in domestic ER or doctor visits (Food and Drug Authority, 2009). In 2011, Advisory Committee on Immunization Practices (ACIP) updated the recommendations for HPV vaccination to include boys, which could have led to an increase in vaccinations and associated ER visits (Centers for Disease Control and Prevention, 2011, Hawkins et al., 2021; Lauri E. Lauri E. Markowitz et al., 2007, Markowitz et al., 2012).

Over the last 31 years, domestic and nondomestic hospitalization reports have steadily declined. However, supporting our previous interpretations, domestic data on HPV vaccine showed an increased rate of hospitalization from 2006 to 2015 (Centers for Disease Control and Prevention, 2011, Hawkins et al., 2021; Lauri E. Lauri E. Markowitz et al., 2007, Markowitz et al., 2012). Furthermore, influenza vaccine was associated with a similar increase in 2011, followed by a steady decline. In addition, USFDA approved a new Zoster (Shingrix) vaccine in 2017, which could explain the increase in hospitalizations rate associated with Zoster in the same year (Shah et al., 2019). However, we found no clear cause for the sudden increase in 2015.

Reports of domestic lethal threats increased in 1998. A similar trend occurred with hepatitis B vaccine. This increase may have occurred due to ACIP’s recommendation in 1998 to expand the children vaccination program to include hepatitis B vaccine (Zhao et al., 2011). However, we found a similar increase in reports of nondomestic lethal threats in 1991, when ACIP first recommended hepatitis B vaccines for all infants (Freed et al., 1994).

Domestic disabilities showed a similar trend as lethal threats associated with hepatitis B vaccines in 1991 and 1998 (Freed et al., 1994, Schillie et al., 2013, Zhao et al., 2011). We found another interesting increase in 1999 and a similar pattern associated with Lyme vaccine, which FDA approved in 1998 but its manufacturer has since withdrawn (Nigrovic and Thompson, 2007, Shen et al., 2011). We also found a similar pattern of Zoster-associated disabilities and lethal threat pattern from 2006 to 2015(Shah et al., 2019). In 2005, we found an increase in the administration of anthrax vaccine, which could have occurred due to the EUA’s extension of anthrax-adsorbed vaccine, which USFDA announced that year (FDA, 2005). Moreover, nondomestic reported disabilities show a similar pattern associated with Zoster vaccine (Shah et al., 2019). We found another interesting pattern described by an increase in nondomestic disabilities in 1998, which was associated with a similar pattern in hepatitis B and MMR II vaccines. Wakefield study results may affect the reporting pattern of MMR. The outcome of this study, which was published in 1998, showed that a behavioral and development disorder in children resulted from MMR (Godlee et al., 2011, Sathyanarayana Rao and Andrade, 2011).

In domestic and nondomestic data, pyrexia was the top reported AEFI over the past 30 years. This AEFI is expected as a part of the activated immune response (Hervé et al., 2019). However, in 2021, headache was the top reported AEFI and was mainly associated with COVID-19 vaccines (Cocores and Monteith, 2016, Hervé et al., 2019). In the domestic data, injection site AEFIs were the most common (Hervé et al., 2019). Furthermore, the nondomestic data shows a variety of symptoms, with vomiting being the commonly reported AEFI (Hervé et al., 2019).

This study results of AESIs analysis show that most reported adverse event associated with COVID-19 was myocarditis or pericarditis, however the reported number in this study (191.6 of each 100 000 report) is much higher than what CDC estimated which is 0.48 cases of myocarditis per 100,000 (Witberg et al., 2021). In addition, pulmonary embolism and stroke were among the highest reported AESIs, which largely agree with the existing published data (Barda et al., 2021, Li et al., 2021a, Li et al., 2021b, Patone et al., 2021). However, despite the high incident rate of bell’s palsy and narcolepsy which reported by Li et al, this study results show low rates of these AESIs (Li et al., 2021b).

Results of our study show that most of AEFIs are nonserious and more than 80 % of them appear within first few days following vaccination, which is consistent with results found in other articles (Alguacil-Ramos et al., 2016, Fadare et al., 2019, Mentzer et al., 2018). In addition, male and age group ≤ 5 years were more prone to serious AEFIs in consistent with findings from prior research (Fadare et al., 2019, Harris et al., 2017). These findings could strengthen the argument for VARES's efficient use.

Generally, stimulated reporting is noticeable in this study’s outcomes (Shimabukuro et al., 2015, Varricchio et al., 2004). Yearly patterns were mostly affected by USFDA’s approval of vaccines such as Lyme and HPV vaccines, CDC recommendations, such as administration of H1N1 and DTP vaccines, and ACIP recommendations, such as administration of HPV and HEB B vaccines. In addition, pandemic diseases, such as COVID-19 and H1N1, have a major impact on vaccination pattern (Campbell and Grohskopf, 2018, Centers for Disease Control and Prevention, 2022, Centers for Disease Control and Prevention, 2011, DeStefano, 2008, Fiore et al., 2010, Food and Drug Authority, 2009, Freed et al., 1994, Grohskopf et al., 2021, Hawkins et al., 2021, Jefferies et al., 2011, Lauri E. Markowitz et al., 2007, Markowitz et al., 2012, Ryan Gierke et al., 2021, Shah et al., 2019, Zhao et al., 2011). Finally, public research outcomes, such as in the case of MMR vaccine, have a noticeable impact on vaccination rate (Godlee et al., 2011, Sathyanarayana Rao and Andrade, 2011).

The study has several limitations, given that this is a passive reporting system, and that each report may contain information about several vaccines, we cannot confirm a causal relationship between a vaccine and its associated AEFIs (Shimabukuro et al., 2015, VAERS, 1990, Varricchio et al., 2004). Incomplete reports and underreporting are a noticeable issue in these data sets, which can be seen clearly with serious AEFIs. In addition, most of the nondomestic data are reported by manufacturers to fulfill legal requirements, which could lead to misleading selective reporting and thereby impact nondomestic data more than domestic data (Shimabukuro et al., 2015). Although VAERS uses MedDRA medical terminology, spelling mistakes and duplication (e.g., pyrexia and elevated body temperature) could have a negative impact on the data (VAERS, 1990).

## 5 Conclusion

VAERS is considered an important and useful tool in vaccine safety surveillance. Despite the fact that the majority of its reported AEFIs were non serious, COVID-19 had a large impact on the reporting patterns. Similar pattern was noticed in prior pandemics but with lower numbers. Although, most of its reported AEFIs were non serious, COVID-19 highly affected the reporting, with a similar noticeable patterns of increase reporting during previous pandemics. Regulatory authorities may be able to identify pattern changes in real time by closely monitoring such a system. With addition to clear public misconception about particular vaccine, this facilitates quick and appropriate action during a pandemic. However, the usability of VAERS data depends on users understating this surveillance system’s limitations and knowing how to interpret its results. Governmental regulations, availability of vaccines, and public health recommendations have the largest impact on reporting rates, which could be used to improve VAERS data.

## 6 Disclaimer

The views expressed in this paper are those of the authors and do not necessarily reflect those of the SFDA or its stakeholders. Guaranteeing the accuracy and the validity of the data is a sole responsibility of the research team.

## Declaration of Competing Interest

The authors declare that they have no known competing financial interests or personal relationships that could have appeared to influence the work reported in this paper.
