# Supplementary material for: Vaccine adverse event reporting system (VAERS): Evaluation of 31 years of reports and pandemics’ impact
Source: Saudi Pharm J. 2022 Oct 7;30(12):1725–35. doi: 10.1016/j.jsps.2022.10.001 (PMC9805973; doi:10.1016/j.jsps.2022.10.001)
Supplement: Supplementary data 1 [file mmc1.docx]

**Figure 1:** Number of reports per year

**Figure 2:** Pattern of reported death over years

**Figure 3:** Patterns over years of top 5 vaccines in domestic reported deaths

**Figure 4:** Patterns over years of top 5 vaccines in nondomestic reported death

**Figure 5**: Pattern of ER or doctor visits over years

**Figure 6:** Patterns over years of top 5 vaccines in domestic reported ER or doctor visits

**Figure 7:** Patterns over years of top 5 vaccines in nondomestic reported ER or doctor visits

**Figure 8:**  Pattern of reported hospitalization over years

**Figure 9:** Patterns over years of top 5 vaccines in domestic reported hospitalization

**Figure 10:** Patterns over years of top 5 vaccines in nondomestic reported hospitalization

**Figure 11:** Pattern of reported lethal threat over years

**Figure 12:** Patterns over years of top 5 vaccines in domestic reported lethal threat

**Figure 13:** Patterns over years of top 5 vaccines in nondomestic reported lethal threat

**Figure 14:** Pattern of reported disabilities over years

**Figure 15:** Patterns over years of top 5 vaccines in domestic reported disabilities

**Figure 16:** Patterns over years of top 5 vaccines in nondomestic reported disabilities
